# PECAM‐1 modulates liver damage induced by synergistic effects of TNF‐α and irradiation

**DOI:** 10.1111/jcmm.14224

**Published:** 2019-02-14

**Authors:** Gesa Malik, Jörg Wilting, Clemens Friedrich Hess, Giuliano Ramadori, Ihtzaz Ahmed Malik

**Affiliations:** ^1^ Clinic for Gastroenterology and Endocrinology University Medical Center Göttingen Göttingen Germany; ^2^ Department of Anatomy and Cell Biology University Medical Center Göttingen Göttingen Germany; ^3^ Clinic for Radiotherapy and Radiooncology University Medical Center Göttingen Göttingen Germany

**Keywords:** CD31, cytokines, inflammation, lipocalin-2, RILD

## Abstract

The mechanisms of radiation‐induced liver damage are poorly understood. We investigated if tumour necrosis factor (TNF)‐α acts synergistically with irradiation, and how its activity is influenced by platelet endothelial cell adhesion molecule‐1 (PECAM‐1). We studied murine models of selective single‐dose (25 Gy) liver irradiation with and without TNF‐α application (2 μg/mouse; i.p.). In serum of wild‐type (wt)‐mice, irradiation induced a mild increase in hepatic damage marker aspartate aminotransferase (AST) in comparison to sham‐irradiated controls. AST levels further increased in mice treated with both irradiation and TNF‐α. Accordingly, elevated numbers of leucocytes and increased expression of the macrophage marker CD68 were observed in the liver of these mice. In parallel to hepatic damage, a consecutive decrease in expression of hepatic PECAM‐1 was found in mice that received radiation or TNF‐α treatment alone. The combination of radiation and TNF‐α induced an additional significant decline of PECAM‐1. Furthermore, increased expression of hepatic lipocalin‐2 (LCN‐2), a hepatoprotective protein, was detected at mRNA and protein levels after irradiation or TNF‐α treatment alone and the combination of both. Signal transducer and activator of transcription‐3 (STAT‐3) seems to be involved in the signalling cascade. To study the involvement of PECAM‐1 in hepatic damage more deeply, the liver of both wt‐ and PECAM‐1‐knock‐out‐mice were selectively irradiated (25 Gy). Thereby, ko‐mice showed higher liver damage as revealed by elevated AST levels, but also increased hepatoprotective LCN‐2 expression. Our studies show that TNF‐α has a pivotal role in radiation‐induced hepatic damage. It acts in concert with irradiation and its activity is modulated by PECAM‐1, which mediates pro‐ and anti‐inflammatory signalling.

## 1 INTRODUCTION

Irradiation is one of the current treatment options for cancer. With the enormous progress in this field, it became technically possible to selectively irradiate most types of tumours. However, one of the shortcomings of this procedure is the unwanted effect on healthy tissues adjacent to the tumour. In the liver, a limiting factor for irradiation is radiation‐induced liver disease (RILD) also known as radiation hepatitis. RILD is regarded as a major limiting factor for radiotherapy of primary and secondary liver tumours.^1^, ^2^ Therefore, the identification of molecular interactions involved in RILD may help to improve radiotherapeutic options, for example by the protection of non‐diseased tissue from unwanted side effects of irradiation. Furthermore, the irradiation of liver tumours is often performed in patients with additional local or general diseases, which may increase radiation toxicity.^3^ Of note, our in vitro studies have recently shown that normal liver cells are highly resistant against radiation,^4^ which appears to be in contrast to the high incidence of RILD. However, combinatory effects of tumour necrosis factor (TNF)‐α application and radiation increase radiosensitivity in healthy liver cells, associated with the release of mediators of cellular injury.^5^, ^6^, ^7^

Tissue damage outside or within the liver leads to the induction of acute‐phase (AP)‐response, which is an early defense mechanism required to maintain homeostasis and initiate repair. Clinically, AP‐response is characterized by fever, anorexia, as well as change or dramatic increase in concentration of serum proteins (positive AP‐proteins). Thereby, release of positive AP‐proteins such as C‐reactive protein (CRP) in humans, or lipocalin‐2 (LCN‐2) and serum amyloid A (SAA) in mice, subsequent to pro‐inflammatory cytokines, is a hallmark of AP‐reaction. TNF‐α is one of the major AP‐cytokines.^8^ It is well known for its role in cell injury probably through the generation of reactive oxygen species (ROS)^7^, ^9^, ^10^ or apoptosis.^11^ In liver, an active involvement of TNF‐α in both acute and chronic liver inflammation has extensively been investigated.^7^, ^10^, ^12^ Kupffer cells are the main source for TNF‐α, but it is also produced by other liver cells during stress conditions, such as hepatocytes.^13^, ^14^ TNF‐α levels have been reported to be elevated in viral hepatitis, alcoholic and non‐alcoholic fatty liver disease and liver injury in humans and rodents.^7^, ^15^, ^16^, ^17^ Accordingly, we observed elevated TNF‐α levels in various animal models of selective liver irradiation^4^, ^18^ and acute toxin‐induced liver damage.^17^

Several reports have demonstrated a positive correlation between tissue damage and the immigration of inflammatory cells.^7^, ^19^, ^20^, ^21^ Thereby, TNF‐α, which is of major importance for the maturation of humoural immune response, also regulates leucocyte transmigration via adhesion molecules and inflammatory mediators.^22^, ^23^ PECAM‐1/CD31 is an adhesion molecule constitutively expressed by endothelial cells, macrophages, neutrophils and lymphocytes.^23^, ^24^ Initially, the role of PECAM‐1 in inflammation was considered to be a minor one and was controversially debated. Moreover, the down‐regulation of PECAM‐1, both at the endothelial and the leucocyte surface, was considered to be a consequence of transmigration of leucocytes through the vessel wall and not the pre‐condition for their transmigration. In fact, PECAM‐1 down‐regulation can be induced by cytokines in vitro without the need for contact between endothelial cells and leucocytes.^23^, ^25^ However, in recent years, data supporting its possible role as ‘anti‐inflammatory’ adhesion molecule are increasing.^19^, ^24^ Lack of PECAM‐1 is associated with greater tissue damage in a model of acute inflammation induced by lipopolysaccharides (LPS).^19^, ^26^ In line with this, we have recently shown that PECAM‐1‐ko mice are more sensitive to irradiation, which is associated with increased production of inflammatory mediators, especially TNF‐α.^4^ Therefore, the current study investigated the probability of synergistic effects of TNF‐α and irradiation in liver damage and the potential role of PECAM‐1.

## 2 MATERIALS AND METHODS

### 2.1 Materials

All chemicals and reagents were purchased from commercial sources Sigma‐Aldrich (St. Louis, USA) or Merck (Darmstadt, Germany).

### 2.2 Animal models

All mice used in this study were male and 8‐12 weeks old with a body weight of 20‐28 g. Mice were purchased from The Jackson Laboratory (Bar Harbor, ME, USA). Wild‐type (wt) (C57BL/6J) mice received single‐dose selective liver irradiation (25 Gy, dose rate 2.4 Gy/min) with or w/o injection of TNF‐α (2 μg/mouse) (Roche, Penzberg, Germany). A single injection of TNF‐α was administered intraperitoneally (i.p.) 20 minutes before irradiation, as described previously.^27^ PECAM‐1‐knock‐out (ko) (B6.129S‐*Pecam1* ^*Gt*(*OST1630*3*)Lex*^/J) mice also received selective liver irradiation with a single dose of 25 Gy as described before.^4^ The animals were killed at regular intervals at 1‐48 hours after irradiation. Sham‐irradiated and PBS‐injected (i.p.) control animals (n = 3 per each time‐point) were studied simultaneously in all experiments.

All animal studies were reviewed and approved by the committee of the Central Institute for Animal Experiments of the University Medical Centre Goettingen, and the Lower Saxony State Office for Consumer Protection and Food Safety (No. 33.12‐42502‐04‐10/0158).

### 2.3 Measurement of aspartate aminotransferase (AST) in murine serum

At regular intervals between 1 and 48 hours following selective liver irradiation, blood samples from the *V. cava inferior* were collected from irradiated and sham‐irradiated mice and used for AST measurement using analysis kits (DiaSys Deutschland, Flacht, Germany) according to the suppliers instructions.

### 2.4 Immunofluorescence double‐staining of mice liver sections

Immunofluorescence staining was performed as described before.^4^, ^28^ Briefly, for double‐staining, monoclonal F4/80 antibody (Abcam, Cambridge, UK; dilution 1:10) was co‐incubated with polyclonal antibody directed against cytokeratin 19 (CK19) (Abcam; dilution 1:50). Liver cryosections of 5 μm thickness were fixed with cold acetone/methanol, washed with PBS, and incubated with blocking medium (0.1% BSA, 10% FCS in PBS) for 1 hour. Sections were incubated with primary antibody at 4°C overnight. Non‐immune serum served as negative control. Secondary antibodies were purchased from Molecular Probes (Leiden, The Netherlands; dilution 1:400). DAPI (4′,6‐diamidino‐2‐phenylindole; Southern Biotech, Birmingham, USA) was used for nuclear counterstaining. The stained sections were investigated with an Axiovert 200M epifluorescence microscope (Zeiss, Jena, Germany).

### 2.5 RNA isolation and real‐time PCR analysis

Total RNA from the livers of irradiated and sham‐irradiated mice was isolated after homogenization in Trizol (Invitrogen, Carlsbad, USA) as described previously.^4^ q‐RT‐PCR was performed with cDNA as described previously with primers (Invitrogen) listed in Table 1.

**Table 1:** Mice primer sequences used in this study

Primer	5–3Forward	5–3Reverse
PECAM‐1	AACAGAAACCCGTGGAGATG	GTCTCTGTGGCTCTCGTTCC
LCN‐2	AAATTGCACAGGTATCCTCAG	CAGAGAAGATGATGTTGTCGT
CD68	CCAGCTGTTCACCTTGACCT	TCACGGTTGCAAGAGAAACA
β‐actin	ATTGTTACCAACTGGGACGACATG	CGAAGTCTAGAGCAACATAGCACA
GAPDH	AGAACATCATCCCTGCATCC	CACATTGGGGGTAGGAACAC

### 2.6 Protein extraction and Western blot analysis

Protein extraction and Western blot analysis were performed as described before.^23^ Briefly, 50 μg of protein was loaded on polyacrylamide gels (NuPAGE 4%‐12% Bis‐Tris Gel, Invitrogen, Carlsbad, CA, USA) under reducing conditions. After electrophoresis, proteins were transferred to Hybond‐ECL (enhanced chemiluminescence) nitrocellulose membranes. Immunodetection was carried out as described previously 23 with antibodies and concentrations listed in Table 2.

**Table 2:** Antibodies used in the study

Antibodies	Company	Reference number	WB	IHC
Rb to CK19	Abcam	Ab133496	–	1:100
Rat anti F4/80 (BM8)	Abcam	Ab16911	–	1:10
Mouse anti Phospho‐STAT3	Cell signaling	#4113S	1:1000	–
Beta‐actinMs anti ß‐Actin Clone AC 15	Sigma	A‐5441	1:5000	_
Mouse monoclonal anti LCN2	Acris	NBP1‐05182	1‐300	

### 2.7 Statistical analysis

The data were analysed using GraphPad Prism version 4 software (San Diego, USA). All experimental errors are shown as SEM. Statistical significance was calculated by Student's *t* test. Significant differences were considered as *P* ≤ 0.05 [* or ^#^], *P* ≤ 0.01 [** or *##*], and *P* ≤ 0.001 [*** or *###*].

## 3 RESULTS

### 3.1 Elevated aspartate aminotransferase levels in serum after irradiation of wt‐mice

**Figure 1:**
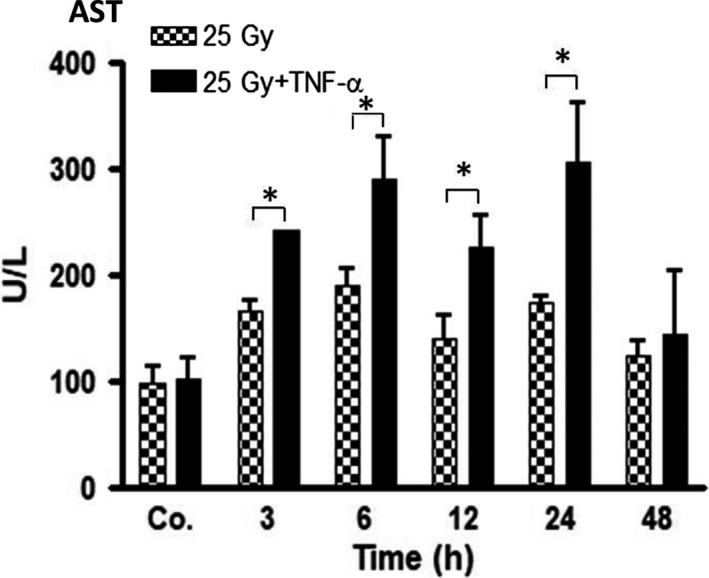
Aspartate aminotransferase (AST) concentrations in serum of wt‐mice at regular time‐points (3‐48 hours) after liver irradiation (25 Gy) with and without TNF‐α as compared to controls (Co. = average of controls of each time‐point), which received both PBS (i.p.) and sham‐irradiation. Results represent the mean ± SEM of three to five experiments

To examine liver damage, serum levels of aspartate aminotransferase (AST) were analysed after irradiation with and w/o injection of TNF‐α in wt‐mice. Serum levels of AST were significantly increased in both groups in comparison to sham‐treated controls. AST levels started to rise after 3 hours, and remained elevated until 24 hours in mice that received both irradiation and TNF‐α. The maximum enzyme activity was detected at 24 hours (305 ± 55 U/L). Irradiation‐only induced maximum AST levels at 6 hours (188 ± 16 U/L) compared to sham‐irradiated mice (96 ± 16 U/L). Of note, there was a significant difference between mice that received TNF‐α immediately before irradiation compared to treatment with irradiation‐only (Figure 1).

### 3.2 Changes in CD68 expression and recruitment of leucocytes into liver of wt‐mice

**Figure 2:**
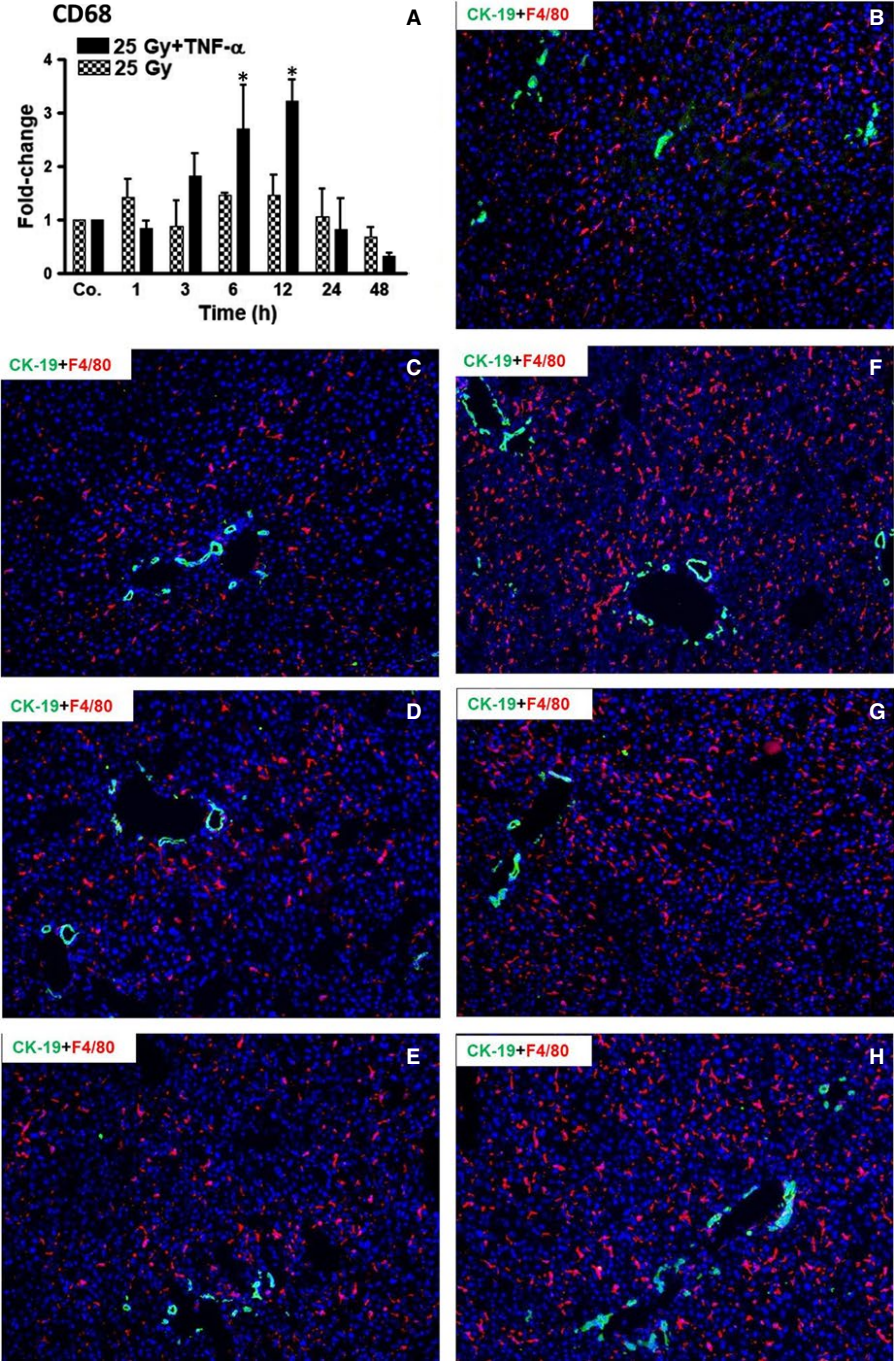
(A) qRT‐PCR analysis from total RNA in liver after irradiation (25 Gy) with and without TNF‐α at various time‐points (1‐48 hours), compared to sham‐irradiated controls (Co.), which received both PBS (i.p.) and sham‐irradiation. Fold‐change in mRNA expression of CD68 is shown. qRT‐PCR was normalized using two housekeeping genes: β‐actin and GAPDH. Results represent mean values ± SEM of each five animals. Note the significant effects of TNF‐α after 6 and 12 hours. * = Comparison irradiation (25 Gy) vs irradiation + TNF‐α treatment. (B‐H) Detection of CK‐19 (green, marker for biliary cells) and F4/80 (red, marker for macrophages) by double immunofluorescence staining in murine liver after irradiation (25 Gy) in the presence/absence of TNF‐α, compared to sham‐irradiated controls. (B) Liver of control mice; (C) 6 hours, (D) 12 hours, (E) 24 hours after irradiation. (F) 6 hours, (G) 12 hours, (H) 24 hours after TNF‐α plus irradiation. Nuclei were stained with DAPI (blue). Note the significantly larger amounts of macrophages in TNF‐α‐treated specimens, which is most obvious after 6 and 12 hours. Original magnification ×100

RNA expression in liver of wt‐mice was analysed by qRT‐PCR and revealed an increase in CD68 expression (indicating higher numbers of macrophages and granulocytes) after combined administration of TNF‐α and irradiation as compared to irradiation‐only. mRNA expression of CD68 was significantly increased 3 hours after the combined administration of TNF‐α and irradiation with a further significant increase at 6 hours (2.68 ± 0.8‐folds) and 12 hours (3.22 ± 0.4‐folds). The level of CD68 decreased thereafter (Figure 2A).

Our qRT‐PCR data were further confirmed by immunofluorescence studies. F4/80^+^ cells (marker for macrophages/Kupffer cells) and CK‐19^+^ cells (marker of biliary cells) were regularly detected in liver of sham‐irradiated mice (Figure 2B). Consistent with our qRT‐PCR‐data, an increase in the number of F4/80^+^ cells was detected after 6 hours in mice treated with TNF‐α plus irradiation, and remained high at 12 and 24 hours. At 48 hours, the number of macrophages decreased to normal levels (data not shown). Notably, no obvious increase in F4/80^+^ cells was detected in murine liver after sole irradiation at any time‐point compared to sham‐irradiated mice (Figure 2B‐G).

### 3.3 Influence of irradiation on PECAM‐1 expression in liver of wt‐mice

**Figure 3:**
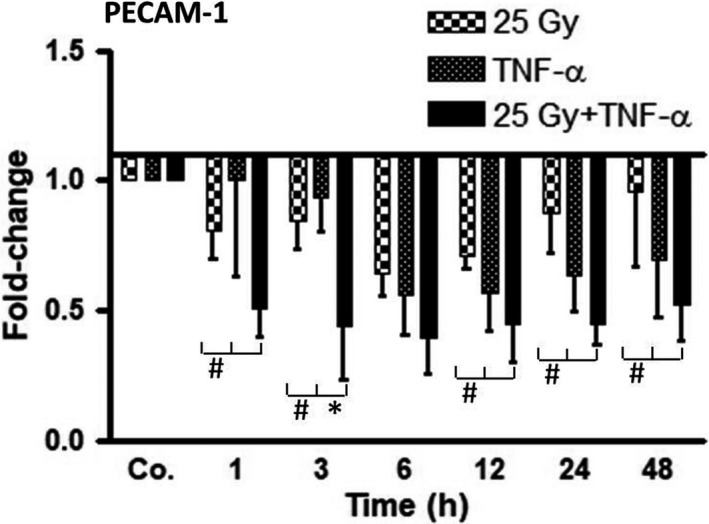
qRT‐PCR analysis from total liver RNA after irradiation (25 Gy), TNF‐α treatment, or combination of both treatments, at various time‐points (1‐48 hours) as compared to controls (Co.), which received both PBS (i.p.) and sham‐irradiation. Fold‐change of mRNA expression of PECAM‐1 is depicted. qRT‐PCR was normalized using two housekeeping genes: β‐actin and GAPDH. Results represent mean values ± SEM for each five animals. Note the significant down‐regulation of PECAM‐1 by radiation plus TNF‐α treatment. * = Comparison irradiation (25 Gy) vs irradiation + TNF‐α treatment. ^#^ = Comparison TNF‐α treatment vs irradiation (25 Gy) + TNF‐α treatment

Total RNA extracted from the livers of wt‐mice treated by irradiation, TNF‐α or both was analysed by real‐time PCR. After irradiation, PECAM‐1‐specific transcripts started to decrease early (1 hour) and reached a minimum after 6 hours (0.64 ± 0.1‐fold) as compared to control mice (Figure 3). Mice treated with only TNF‐α showed a similar pattern of decrease in PECAM‐1, with an expression minimum at 6 hours (0.56 ± 0.1‐fold), which remained low until 48 hours. After selective liver irradiation or TNF‐α alone, the expression levels of PECAM‐1 remained below the values of control mice until 48 hours. Importantly, the administration of irradiation together with TNF‐α additionally reduced the PECAM‐1 expression (6 hours; 0.39 ± 0.14‐fold) as compared to each single treatment; and levels remained significantly low until 48 hours (Figure 3).

### 3.4 Kinetics of LCN2 in liver of wt‐mice after irradiation and TNF‐α

**Figure 4:**
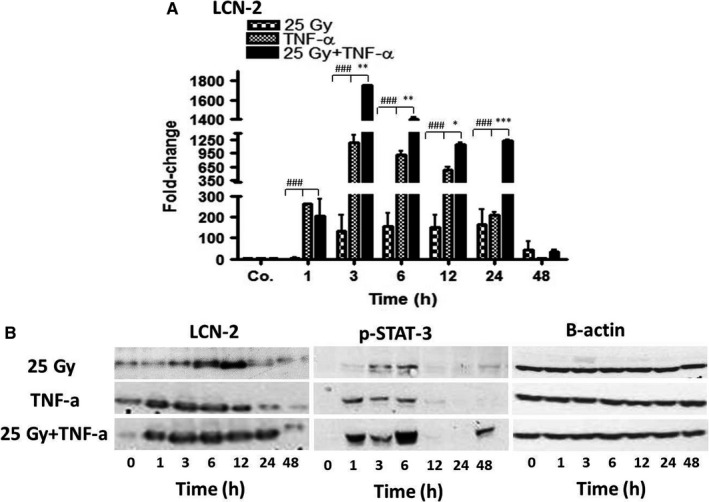
qRT‐PCR and Western blot analyses from total RNA and protein in liver after irradiation (25 Gy), TNF‐α treatment, or combination of both, at various time‐points (1‐48 hours) as compared to controls (Co.), which received both PBS (i.p.) and sham‐irradiation. (A) Fold‐change of mRNA expression of LCN‐2. qRT‐PCR was normalized using two housekeeping genes: β‐actin and GAPDH. Results represent mean value ± SEM of each five animals. * = Comparison irradiation (25Gy) vs irradiation + TNF‐α treatment. ^#^ = Comparison TNF‐α vs irradiation (25 Gy) + TNF‐α treatment. (B) Western blot analyses of protein from mouse liver with antibodies against LCN‐2 (left), pSTAT‐3 (middle), and β‐actin (right) as a loading control. Results are representative of three experiments

In contrast to PECAM‐1, the expression of LCN‐2 was increased in the liver of wt‐mice after single‐dose selective liver irradiation with or w/o TNF‐α injection at RNA and protein level as compared to sham‐irradiated controls. The level of LCN‐2 started to increase immediately (1 hour) after irradiation (4 ± 2‐fold), further increased after 3 hours, remained high after 12 hours (163 ± 74‐fold) and 24 hours, and decreased thereafter (Figure 4A). More efficiently, TNF‐α administration alone induced RNA expression of LCN‐2, with a dramatic increase after 1 hour (263 ± 1‐fold) and reached a peak value after 3 hours (1190 ± 180‐fold). The levels of LCN‐2 remained elevated until 24 hours and decreased thereafter. A dramatic induction in LCN‐2 RNA expression was observed in mice who received irradiation in combination with TNF‐α. The combined treatment raised LCN‐2 expression after 1 hour (203 ± 80‐fold), followed by a massive induction at 3 hours (2149 ± 553‐fold). Then, the expression of LCN‐2 started to decrease but remained drastically elevated even at 24 hours (1237 ± 29‐fold). Thereby, from 3 to 24 hours, LCN‐2 expression after combination treatment was significantly higher than each single treatment (Figure 4A). Importantly, qRT‐PCR results could be confirmed at protein level by Western blotting (Figure 4B).

### 3.5 Phosphorylation of STAT‐3 in the liver of wt‐mice after irradiation and TNF‐α

Signal transducer and activator of transcription‐3 (STAT‐3) is an important transcription factor in inflammatory signalling pathways. By Western blot analysis with specific antibodies against pSTAT‐3, phosphorylation of STAT‐3 was noticed at 3 and 6 hours in mice after irradiation (Figure 4B). A similar result was observed in mice who received TNF‐α only. There, phosphorylation already occurred after 1 hour. Of note, the most intense signals of pSTAT‐3 were visible in mice who received radiation plus TNF‐α. The strength of this band increased further at 6 hours, and rapidly decreased thereafter. Of note, the magnitude of phosphorylation was the highest in mice, who received both irradiation and TNF‐α (Figure 4B).

### 3.6 Detection of the liver damage in wt and PECAM‐1‐ko‐mice after irradiation

**Figure 5:**
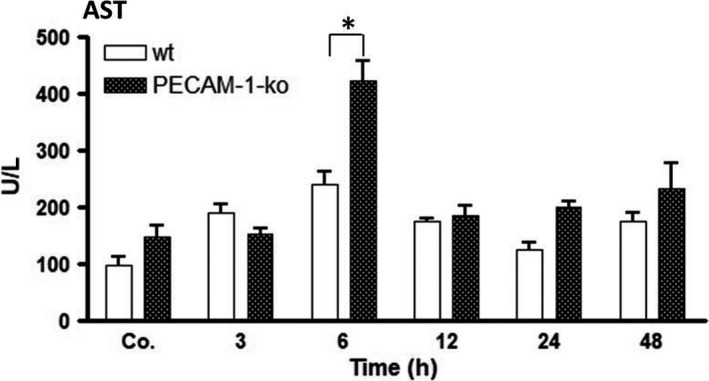
Aspartate aminotransferase (AST) concentrations in serum of wt and PECAM‐1‐ko‐mice at various time‐points (3‐48 hours) after irradiation, as compared to sham‐irradiated controls (Co. = average of controls of each time‐point). The data are compared between wt and ko‐mice and with sham‐irradiated controls. Results represent the mean ± SEM of three to five experiments

Liver damage after irradiation was confirmed by measuring the serum levels of AST both in wt and PECAM‐1‐ko‐mice. Both groups of mice showed an increase in AST as compared to sham‐irradiated controls (Figure 5). The levels of AST rose immediately after irradiation with a maximum at 6 hours (238 ± 22 U/L). However, there was a significant difference between wt (238 ± 22 U/L) and ko‐mice (422 ± 33 U/L) at this time‐point, the ko‐mice showing significantly higher levels of the enzyme. AST levels then decreased (Figure 5).

### 3.7 Changes in level of hepatic LCN‐2 after irradiation

**Figure 6:**
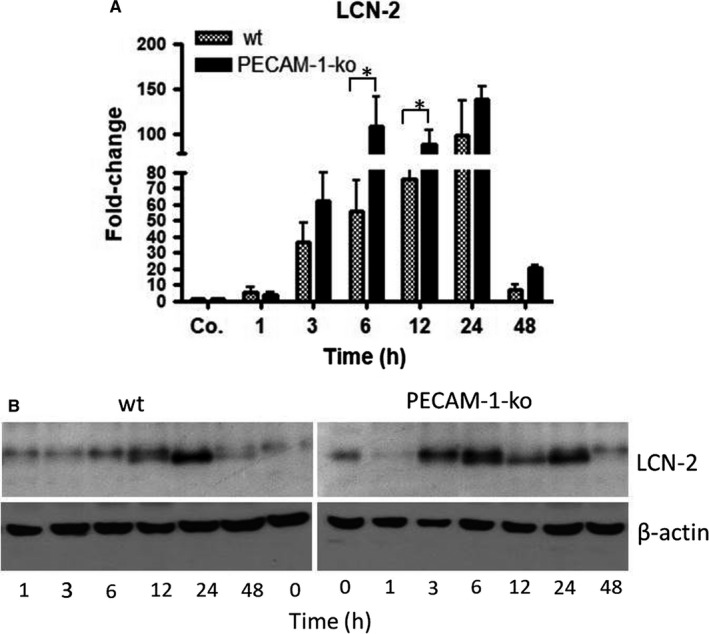
(A) Fold‐change of mRNA expression of LCN‐2 in the liver of wt and PECAM‐1‐ko mice at various time‐points after single‐dose liver irradiation compared to controls (Co.). RT‐PCR was normalized to β‐actin and GAPDH. Results represent mean ± SEM values of four to six experiments (in duplicate) compared to controls for each time‐point. (B) Western blot analysis of total protein from liver of wild‐type (wt) and PECAM‐1‐ko‐mice at various time‐points after liver irradiation. β‐actin (~42 kDa) was used as a loading control. Results are representative of four experiments

To validate the role of PECAM‐1 in radiation‐induced liver stress, we analysed the expression of hepatic LCN‐2 in wt and PECAM‐1‐ko‐mice. qRT‐PCR and Western blot analyses showed a time‐dependent increase in LCN‐2 both in wt and PECAM‐1‐ko‐mice after irradiation in comparison to sham‐irradiated controls. An early increase (3 hours) in LCN‐2 was detected in ko‐mice, whereas this increase was visible with a delay (6 hours) in wt‐mice (Figure 6). LCN‐2 reached its maximum at 24 hours (99 ± 37‐fold and 139 ± 14‐fold in wt and ko‐mice respectively). Thereby, the magnitude of LCN‐2 expression was significantly higher in ko‐mice than in wt‐mice (Figure 6A,B), indicating increased hepatoprotective mechanisms in PECAM‐1‐null‐mice.

## 4 DISCUSSION

### 4.1 TNF‐α and radiation‐induced liver disease

The use of radiation therapy may be extended to hepatic cancers (primary and metastatic) if local side effects of radiation could be controlled more efficiently. Although technical progress has made it possible to irradiate tumours with high accuracy, damage of non‐tumoural tissue still remains a major limitation. Radiobiologists are currently investigating the mechanisms of RILD; however, the availability and validity of radiation models which recapitulate RILD conditions are scarce. As the majority of studies are performed in tumour‐bearing animal models or tumour cell lines, the mechanisms of radiation‐induced liver damage in healthy liver have remained poorly understood. We have started to investigate the impact of irradiation on healthy liver. Our in vitro and in vivo studies clearly demonstrate that healthy liver is highly resistant to irradiation.^4^, ^28^, ^29^ The reason why hepatic cancer patients seem to be hyper‐sensitive to irradiation may be because of the administration of irradiation to diseased liver or administration of chemotherapeutics together with irradiation. We have previously reported that the intracellular defense system of hepatocytes is weakened when irradiation is administered in combination with inflammatory mediators such as TNF‐α.^5^

TNF‐α, when released into the extracellular matrix together with other mediators of cellular noxae, initiates inflammatory processes followed by the recruitment of leucocytes. It is now well‐accepted that immigration of inflammatory cells plays an important role in tissue injury, as has been shown in animal models of toxicity.^20^, ^21^ We previously showed that selective liver irradiation results in cytokine and chemokine expression, mainly TNF‐α and MCP‐1.^4^, ^28^ Their increased synthesis is accompanied by the immigration of distinct numbers of granulocytes, but not mononuclear phagocytes, into the liver and induces an immediately reversible mild hepatic damage.^28^ In the current study, we demonstrate synergistic effects of irradiation and TNF‐α application, which results in considerable numbers of mononuclear phagocytes in liver, in addition to granulocytes, which is in line with previous studies where an accumulation of inflammatory cells is shown to be associated with tissue damage.^28^ This suggests an increased level of liver damage as compared to sole irradiation by increased numbers of newly recruited leucocytes. Similarly, in a rat model of thioacetamide (TAA)‐induced acute liver damage an amplified release of TNF‐α was followed by immigration of both granulocytes and mononuclear phagocytes, which was associated with significant liver damage.^30^

### 4.2 Adhesion molecules and liver irradiation

In addition to cytokines and chemokines, adhesion molecules play a crucial role in the activation of infiltrating cells into liver, their attachment to endothelial cells, primarily those of the portal vessels, and their transmigration towards stressed hepatocytes.^31^, ^32^, ^33^, ^34^ Thereby, TNF‐α‐mediated down‐regulation of PECAM‐1/CD31 (together with up‐regulation of ICAM‐1, not shown) seems to be of great importance for the transmigration and activation of inflammatory cells.^22^, ^23^, ^31^, ^34^ In the current study, we examined the significance of TNF‐α‐induced signalling for (down)‐regulation of PECAM‐1. We and others could evidently show the functional relevance of TNF‐α for the regulation of PECAM‐1 in inflammatory processes,^24^, ^34^ which was considered to be of minor importance previously. Here we show that lack or reduction of PECAM‐1 is associated with elevated and prolonged liver damage as shown by AST serum levels. This is in accordance with other reports, where lack or reduction of PECAM‐1 expression corresponded with greater liver damage after administration of CCl_4_ to animals.^34^ In the same lines, an enhanced liver damage was reported in PECAM‐1‐ko‐mice after LPS exposure, indicating clearly that lack of PECAM‐1 can enhance acute liver damage.^19^, ^26^

It was shown that both IFN‐γ and TNF‐α can down‐regulate PECAM‐1 in various cell types, such as liver endothelial cells, sinusoidal macrophages, peripheral blood leucocytes (PBLs) and granulocytes.^22^, ^23^ Furthermore, the administration of anti‐TNF‐α antibodies (Infliximab) reverses the reduction caused by either IFN‐γ or TNF‐α in PBLs, macrophages and granulocytes.^23^ Anti‐TNF‐α therapy exerts its anti‐inflammatory effects by neutralizing soluble TNF‐α and consequently blocking IFN‐γ signalling.^27^, ^35^ Previously, we have shown that IFN‐γ levels do not change after selective liver irradiation; however, TNF‐α levels increase in both serum and liver, while hepatic PECAM‐1 levels decrease.^4^, ^28^ Our current studies are in concert with previous in vitro data and further show that irradiation‐induced TNF‐α may cause tissue damage by recruiting inflammatory cells through transient down‐regulation of PECAM‐1 in endothelial cells. This effect is enhanced when irradiation and TNF‐α are administered simultaneously to mice. The combined application decreases PECAM‐1 levels in parallel to increased tissue damage. These results were further confirmed in PECAM‐1‐ko‐mice where significantly higher AST levels were observed compared to wt‐mice.

### 4.3 Hepatoprotective mechanisms and liver irradiation

Lipocalin‐2 (LCN‐2) is not only a marker for irradiation‐induced damage but also a positive acute‐phase protein.^36^ It is up‐regulated during hepatic damage, exerts anti‐oxidative effects and acts as a hepatoprotective agent. Thus, the extent of the expression of hepatoprotective agents seems to correlate positively with the amount of liver damage. We observed that combined irradiation and TNF‐α administration further increases the level of LCN‐2 in parallel to tissue injury, indicating that tissue damage is immediately counteracted by protective mechanisms. LCN‐2 induction seems to be regulated by, or at least correlates with, PECAM‐1 as the lack of PECAM‐1 enhances the levels of LCN‐2. Intracellularly, the STAT‐3 pathway may be involved in hepatoprotection. STAT‐3 has a central position in the regulation of cell growth and apoptosis, as well as the systemic regulation of inflammation and cachexia.^37^ In fact, regulation of LCN‐2 through STAT‐3, and the liver protective functions of this pathway, has been well documented.^38^, ^39^, ^40^

Taken together, our studies give insight into irradiation‐induced liver injury through TNF‐α‐regulated PECAM‐1, which may be important not only for leucocyte transmigration through the vascular wall, but also for the regulation of inflammation per se. Furthermore, we could show that after application of the pro‐inflammatory agent TNF‐α the liver becomes more sensitive to radiation. This may explain the high sensitivity of the liver to radiotherapy in patients, when the diseased/inflamed liver is irradiated and additionally treated with chemotherapeutics. Our results also indicate that anti‐inflammatory treatment may help to prevent RILD and its sequel. Thereby, liver protection might be achieved by PECAM‐1 up‐regulation. Our results point to the possibility of developing therapeutics to reduce radiation‐induced damage in normal tissue, as well as agents that may enhance the effects of radiation in tumours.

## CONFLICT OF INTEREST

The authors declare that no actual or potential conflict‐of‐interest in relation to this article exists.

## References

[1] Munoz‐Schuffenegger P , Ng S , Dawson LA . Radiation‐induced liver toxicity. Semin Radiat Oncol. 2017;27:350‐357. 28865518 10.1016/j.semradonc.2017.04.002

[2] Nabavizadeh N , Mitin T , Dawson LA , et al. Stereotactic body radiotherapy for patients with hepatocellular carcinoma and intermediate grade cirrhosis. Lancet Oncol. 2017;18:e192. 28368254 10.1016/S1470-2045(17)30162-6

[3] Guha C , Kavanagh BD . Hepatic radiation toxicity: avoidance and amelioration. Semin Radiat Oncol. 2011;21:256‐263. 21939854 10.1016/j.semradonc.2011.05.003 PMC3434677

[4] Malik IA , Stange I , Martius G , et al. Role of PECAM‐1 in radiation‐induced liver inflammation. J Cell Mol Med. 2015;19:2441‐2452. 26177067 10.1111/jcmm.12630 PMC4594685

[5] Christiansen H , Saile B , Neubauer‐Saile K , et al. Irradiation leads to susceptibility of hepatocytes to TNF‐alpha mediated apoptosis. Radiother Oncol. 2004;72:291‐296. 15450727 10.1016/j.radonc.2004.07.001

[6] Qesaraku B , Dudas J , Rave‐Fränk M , et al. Effect of tumour necrosis factor‐alpha and irradiation alone or in combination on the viability of hepatocellular and biliary adenocarcinoma cell lines in vitro. Liver Int. 2009;29:910‐921. 19226333 10.1111/j.1478-3231.2009.01980.x

[7] Schwabe RF , Brenner DA . Mechanisms of liver injury. I. TNF‐alpha‐induced liver injury: role of IKK, JNK, and ROS pathways. Am J Physiol Gastrointest Liver Physiol. 2006;290:G583‐G589. 16537970 10.1152/ajpgi.00422.2005

[8] Ramadori G , Van Damme J , Rieder H , et al. Interleukin 6, the third mediator of acute‐phase reaction, modulates hepatic protein synthesis in human and mouse. Comparison with interleukin 1 beta and tumor necrosis factor‐alpha. Eur J Immunol. 1988;18:1259‐1264. 3138137 10.1002/eji.1830180817

[9] Pinna F , Bissinger M , Beuke K , et al. A20/TNFAIP3 discriminates tumor necrosis factor (TNF)‐Induced NF‐κB from JNK pathway activation in *Hepatocytes* . Front Physiol. 2017;8:610. 28878689 10.3389/fphys.2017.00610 PMC5572400

[10] Wajant H , Pfizenmaier K , Scheurich P . Tumor necrosis factor signaling. Cell Death Differ. 2003;10:45‐65. 12655295 10.1038/sj.cdd.4401189

[11] Van Antwerp DJ , Martin SJ , Verma IM , et al. Green DR. Inhibition of TNF‐induced apoptosis by NF‐kappa B. Trends Cell Biol. 1998;8:107‐111. 9695819 10.1016/s0962-8924(97)01215-4

[12] Colletti LM , Remick DG , Burtch GD , et al. Role of tumor necrosis factor‐alpha in the pathophysiologic alterations after hepatic ischemia/reperfusion injury in the rat. J Clin Invest. 1990;85:1936‐1943. 2161433 10.1172/JCI114656 PMC296661

[13] Spencer NY , Zhou W , Li Q , et al. Hepatocytes produce TNF‐α following hypoxia‐reoxygenation and liver ischemia‐reperfusion in a NADPH oxidase‐ and c‐Src‐dependent manner. Am J Physiol Gastrointest Liver Physiol. 2013;305:G84‐G94. 23639811 10.1152/ajpgi.00430.2012 PMC3725690

[14] Ramadori G , Moriconi F , Malik I , et al. Physiology and pathophysiology of liver inflammation, damage and repair. J Physiol Pharmacol. 2008;59(Suppl 1):107‐117. 18802219

[15] Manco M , Marcellini M , Giannone G , et al. Correlation of serum TNF‐alpha levels and histologic liver injury scores in pediatric nonalcoholic fatty liver disease. Am J Clin Pathol. 2007;127:954‐960. 17509993 10.1309/6VJ4DWGYDU0XYJ8Q

[16] Tzeng H‐T , Tsai H‐F , Chyuan I‐T , et al. Tumor necrosis factor‐alpha induced by hepatitis B virus core mediating the immune response for hepatitis B viral clearance in mice model. PLoS ONE. 2014;9:e103008. 25047809 10.1371/journal.pone.0103008 PMC4105421

[17] Amanzada A , Moriconi F , Mansuroglu T , et al. Induction of chemokines and cytokines before neutrophils and macrophage recruitment in different regions of rat liver after TAA administration. Lab Invest. 2014;94:235‐247. 24276236 10.1038/labinvest.2013.134

[18] Christiansen H , Sheikh N , Saile B , et al. x‐Irradiation in rat liver: consequent upregulation of hepcidin and downregulation of hemojuvelin and ferroportin‐1 gene expression. Radiology. 2007;242:189‐197. 17090718 10.1148/radiol.2421060083

[19] Privratsky JR , Tilkens SB , Newman DK , Newman PJ . PECAM‐1 dampens cytokine levels during LPS‐induced endotoxemia by regulating leukocyte trafficking. Life Sci. 2012;90:177‐184. 22119535 10.1016/j.lfs.2011.11.002 PMC3264774

[20] Woolbright BL , Jaeschke H . The impact of sterile inflammation in acute liver injury. J Clin Transl Res. 2017;3:170‐188. 28670626 10.18053/jctres.03.2017S1.003 PMC5488807

[21] Woolbright BL , Jaeschke H . Role of the inflammasome in acetaminophen‐induced liver injury and acute liver failure. J Hepatol. 2017;66:836‐848. 27913221 10.1016/j.jhep.2016.11.017 PMC5362341

[22] Neubauer K , Ritzel A , Saile B , et al. Decrease of platelet‐endothelial cell adhesion molecule 1‐gene‐expression in inflammatory cells and in endothelial cells in the rat liver following CCl(4)‐administration and in vitro after treatment with TNFalpha. Immunol Lett. 2000;74:153‐164. 10996391 10.1016/s0165-2478(00)00203-0

[23] Moriconi F , Malik IA , Amanzada A , et al. The anti‐TNF‐α antibody infliximab indirectly regulates PECAM‐1 gene expression in two models of in vitro blood cell activation. Lab Invest. 2012;92:166‐177. 22042082 10.1038/labinvest.2011.160

[24] Privratsky JR , Newman DK , Newman PJ . PECAM‐1: conflicts of interest in inflammation. Life Sci. 2010;87:69‐82. 20541560 10.1016/j.lfs.2010.06.001 PMC2917326

[25] Privratsky JR , Tourdot BE , Newman DK , et al. The anti‐inflammatory actions of platelet endothelial cell adhesion molecule‐1 do not involve regulation of endothelial cell NF‐kappa B. J Immunol. 2010;184:3157‐3163. 20173029 10.4049/jimmunol.0901944 PMC3628820

[26] Maas M , Stapleton M , Bergom C , et al. Endothelial cell PECAM‐1 confers protection against endotoxic shock. Am J Physiol Heart Circ Physiol. 2005;288:H159‐H164. 15319204 10.1152/ajpheart.00500.2004

[27] Martius G , Cameron S , Rave‐Fränk M , et al. The anti‐TNF‐α antibody infliximab inhibits the expression of fat‐transporter‐protein FAT/CD36 in a selective hepatic‐radiation mouse model. Int J Mol Sci. 2015;16:4682‐4697. 25739082 10.3390/ijms16034682 PMC4394442

[28] Malik IA , Moriconi F , Sheikh N , et al. Single‐dose gamma‐irradiation induces up‐regulation of chemokine gene expression and recruitment of granulocytes into the portal area but not into other regions of rat hepatic tissue. Am J Pathol. 2010;176:1801‐1815. 20185578 10.2353/ajpath.2010.090505 PMC2843471

[29] Moriconi F , Christiansen H , Raddatz D , et al. Effect of radiation on gene expression of rat liver chemokines: in vivo and in vitro studies. Radiat Res. 2008;169:162‐169. 18220462 10.1667/RR1006.1

[30] Malik IA , Wilting J , Ramadori G , et al. Reabsorption of iron into acutely damaged rat liver: a role for ferritins. World J Gastroenterol. 2017;23:7347‐7358. 29151689 10.3748/wjg.v23.i41.7347 PMC5685841

[31] Crockett J , Newman DK , Newman PJ . PECAM‐1 functions as a negative regulator of laminin‐induced platelet activation. J Thromb Haemost. 2010;8:1584‐1593. 20403098 10.1111/j.1538-7836.2010.03883.x PMC2909358

[32] Mamdouh Z , Chen X , Pierini LM , et al. Targeted recycling of PECAM from endothelial surface‐connected compartments during diapedesis. Nature. 2003;421:748‐753. 12610627 10.1038/nature01300

[33] Muller WA . Leukocyte‐endothelial‐cell interactions in leukocyte transmigration and the inflammatory response. Trends Immunol. 2003;24:327‐334. 12810109 10.1016/s1471-4906(03)00117-0

[34] Neubauer K , Lindhorst A , Tron K , et al. Decrease of PECAM‐1‐gene‐expression induced by proinflammatory cytokines IFN‐gamma and IFN‐alpha is reversed by TGF‐beta in sinusoidal endothelial cells and hepatic mononuclear phagocytes. BMC Physiol. 2008;8:9. 18466611 10.1186/1472-6793-8-9 PMC2396664

[35] Martius G , Alwahsh SM , Rave‐Fränk M , et al. Hepatic fat accumulation and regulation of FAT/CD36: an effect of hepatic irradiation. Int J Clin Exp Pathol. 2014;7:5379‐5392. 25197426 PMC4152116

[36] Sultan S , Cameron S , Ahmad S , et al. Serum Lipocalin2 is a potential biomarker of liver irradiation damage. Liver Int. 2013;33:459‐468. 23331620 10.1111/liv.12073

[37] Zimmers TA , Fishel ML , Bonetto A . STAT3 in the systemic inflammation of cancer cachexia. Semin Cell Dev Biol. 2016;54:28‐41. 26860754 10.1016/j.semcdb.2016.02.009 PMC4867234

[38] Borkham‐Kamphorst E , van de Leur E , Zimmermann HW , et al. Protective effects of lipocalin‐2 (LCN2) in acute liver injury suggest a novel function in liver homeostasis. Biochim Biophys Acta. 2013;1832:660‐673. 23376114 10.1016/j.bbadis.2013.01.014

[39] Xu M‐J , Feng D , Wu H , et al. The liver is the major source of elevated serum lipocalin‐2 levels after bacterial infection or partial hepatectomy: a critical role for IL‐6/STAT3. Hepatology. 2015;61:692‐702. 25234944 10.1002/hep.27447 PMC4303493

[40] Liedtke C , Trautwein C . A protective role of Stat3 in Fas mediated apoptosis of the liver. J Hepatol. 2004;40:874‐875. 15094244 10.1016/j.jhep.2004.02.020

